# Evolving mtDNA populations within cells

**DOI:** 10.1042/BST20190238

**Published:** 2019-09-04

**Authors:** Iain G. Johnston, Joerg P. Burgstaller

**Affiliations:** 1 Faculty of Mathematics and Natural Sciences, University of Bergen, Bergen, Norway; 2 Department for Agrobiotechnology, Biotechnology in Animal Production, IFA Tulln, 3430 Tulln, Austria; 3 Institute of Animal Breeding and Genetics, University of Veterinary Medicine Vienna, Veterinärplatz 1, 1210 Vienna, Austria

**Keywords:** heteroplasmy, mitochondria, mtDNA

## Abstract

Mitochondrial DNA (mtDNA) encodes vital respiratory machinery. Populations of mtDNA molecules exist in most eukaryotic cells, subject to replication, degradation, mutation, and other population processes. These processes affect the genetic makeup of cellular mtDNA populations, changing cell-to-cell distributions, means, and variances of mutant mtDNA load over time. As mtDNA mutant load has nonlinear effects on cell functionality, and cell functionality has nonlinear effects on tissue performance, these statistics of cellular mtDNA populations play vital roles in health, disease, and inheritance. This mini review will describe some of the better-known ways in which these populations change over time in different organisms, highlighting the importance of quantitatively understanding both mutant load mean and variance. Due to length constraints, we cannot attempt to be comprehensive but hope to provide useful links to some of the many excellent studies on these topics.

## Introduction

Mitochondria are endosymbiotic organelles that facilitated and continue to support all complex life. Due to their evolutionary history, mitochondria in present-day eukaryotic cells retain highly reduced genomes, which encode genes vital for cellular bioenergetics. Eukaryotic cells may contain hundreds or thousands of mitochondrial DNA (mtDNA) molecules. This mini review will discuss how these cellular mtDNA populations evolve over time, particularly focussing on populations involving a mixture of mtDNA types.

The gene content of mtDNA varies dramatically across life [1,2]. Parasitic organisms typically have highly reduced genomes; some have lost mtDNA altogether, retaining highly reduced mitochondrion-related organelles or MROs [3,4] (which may sometimes retain aerobic capacity [5]). Many bilaterians have similar mtDNA complements, although some diversity in gene content and genome structure certainly exists, and in non-bilaterian animals, this diversity expands [6]. Plants often retain more genes and can have huge mtDNA genomes largely filled with non-coding content [7,8]. The highest mtDNA gene counts yet found are retained in some protists [9]. The reasons for this diversity in gene content remain debated but may involve species- and environment-specific resolutions to an evolutionary tension [7,10] between retaining genes for local convenience [11–13] and transferring them to the nucleus for genetic robustness [14–16].

In addition to this diversity in gene content, mtDNA sequences vary within cells and populations. MtDNA is subject to mutation [17–20]. In animals, mtDNA sequence mutation rates are higher than nuclear mutation rates [17,18]. Plant mtDNA, by contrast, has a lower sequence mutation rate than the nucleus [21]. However, the rate of structural mutation (reorganisation of mtDNA molecules) is high in plants [21–25], while animal mtDNA structure is relatively stable [10,19]. Fungi differ again: while mtDNA recombination is common [26,27] and structural variants frequently arise (including the well-known ‘petite’ mutant, with large deletions and an inability to respire [28]), mtDNA mutation rates remain high relative to the nucleus [17,18].

Given this potential for sequence changes, population histories lead to, for example, geographical variation in mtDNA types. In humans, this variation is used to track population histories [29,30] and is a potentially important source of stratification in personalised medicine [31].

MtDNA is physically contained within mitochondria. Animal and fungal mitochondria have physically flexible forms, undergoing fusion into reticulated networks and fission into smaller fragments, and with each organelle typically containing several copies of mtDNA [32]. These molecules are packaged in nucleoids, the size of which is debated [33–35] but which recent evidence suggests usually contain under two mtDNA molecules [36] and are internally genetically homogeneous [37]. Plant mitochondria usually (with some exceptions [38]) remain more as discrete, dynamic organelles [39–43] and often contain no mtDNA [44].

**Figure 1.**
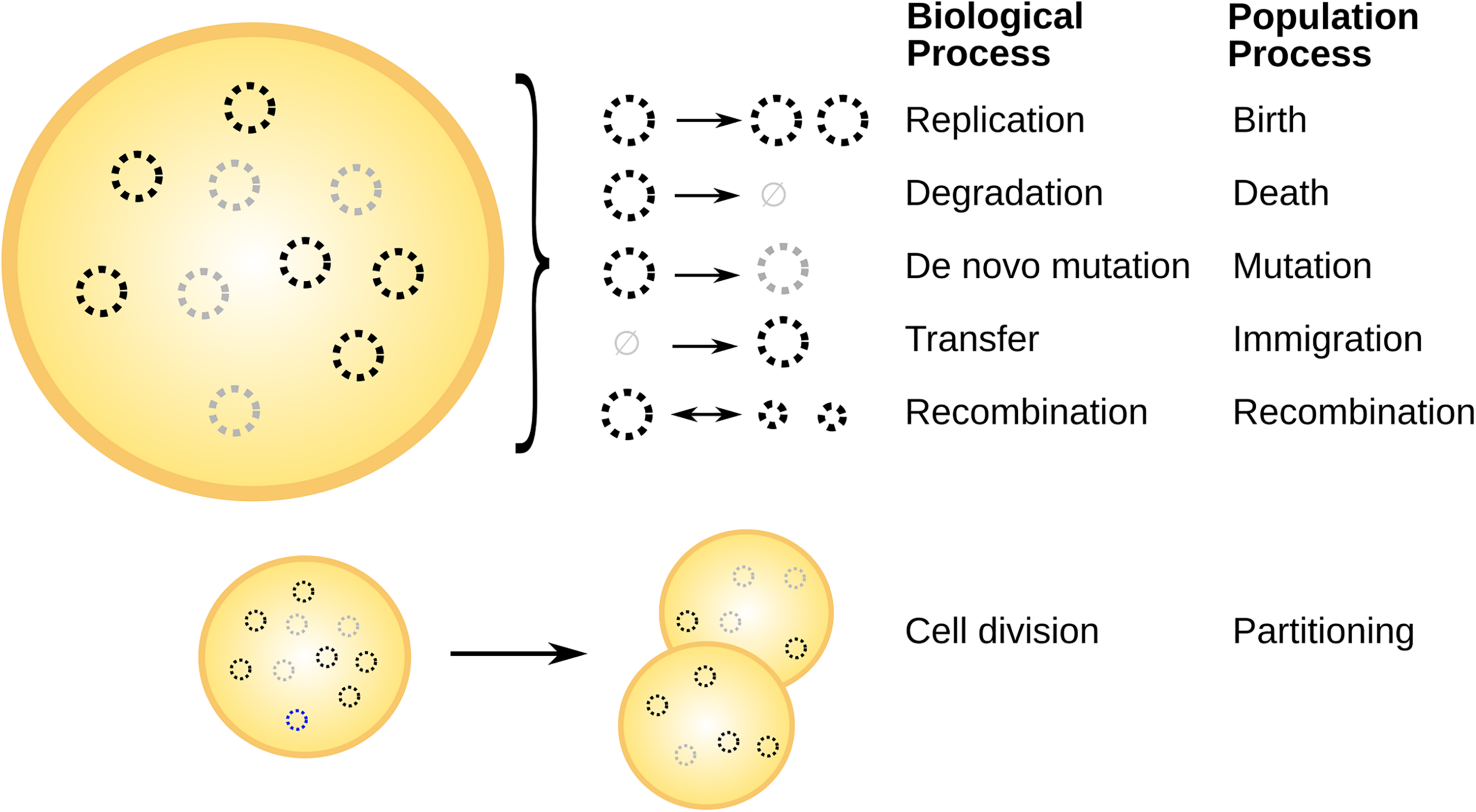
Processes influencing evolving mtDNA populations. Within a heteroplasmic cell containing different types of mtDNA molecule (left), different processes (right) can change the structure of the cellular mtDNA population. These include replication, degradation, *de novo* mutation, intercellular transfer, and recombination. Cell divisions, where mtDNA molecules may be partitioned between daughter cells according to several possible mechanisms, also influence mtDNA statistics. The rates of these processes depend on organism-, sequence-, tissue-, and time-dependent factors. Several correspond directly to processes from the theory of stochastic population processes [57,58].

Within cells, different processes act to dynamically change the structure of mtDNA populations (Figure 1). Across species, mtDNA replication and degradation changes the makeup of the cellular population over time. This is often pictured as ‘relaxed replication’ [45], (replication co-ordinated with, but not directly linked to, the cell cycle [46]), and under nuclear control [47–50]. In animals, mtDNA is largely asexual and exists in reasonably consistent circular forms (with some exceptions, including mtDNA networks in the human heart [51]). In fungi and plants, active recombination mixes and reforms mtDNA content [27,52,53]. This may result in a large variety of branched and linear forms containing different gene content [7,54,55]. The susceptibility of mtDNA to processes including degradation and recombination depends on the physical dynamics of mitochondria, coupling the physical and genetic structure of the mitochondrial population [7,50,56]. *De novo* mutation, and mtDNA transfer between cells, also influence the makeup of mtDNA populations.

The reader will notice the analogy with ecology. Individual mtDNAs exist in cellular ‘ecosystems’, replicating and degrading, mutating, potentially moving between ecosystems, occupying new ground when cells divide, and in some systems also undergoing recombination. The natural question emerges — how heterogeneous are these populations [50]?

### Heteroplasmy

While several mechanisms exist to keep cellular mtDNA populations homogeneous ([59]; see below), sequence and structural differences between mtDNA molecules can result in so-called heteroplasmy, a mixture of different mtDNA variants in the same cell [35,60]. These variants may involve single nucleotide polymorphisms or more dramatic structural changes. Heteroplasmy may emerge from *de novo* mutation, intercellular transfer, recombination, inheritance of different mtDNA types, or synthetically via gene therapies.

Naturally occurring mtDNA heteroplasmy is common across life [61]. Early examples of heteroplasmy were reported in organisms as diverse as fly [62], seaweed [63], fungi [64], maize [65], brittle stars [66], flatfish [67], and vegetatively propagated olive trees [68]. Low-level heteroplasmy is ubiquitous in humans [69] and more broadly across vertebrates [70].

**Figure 2.**
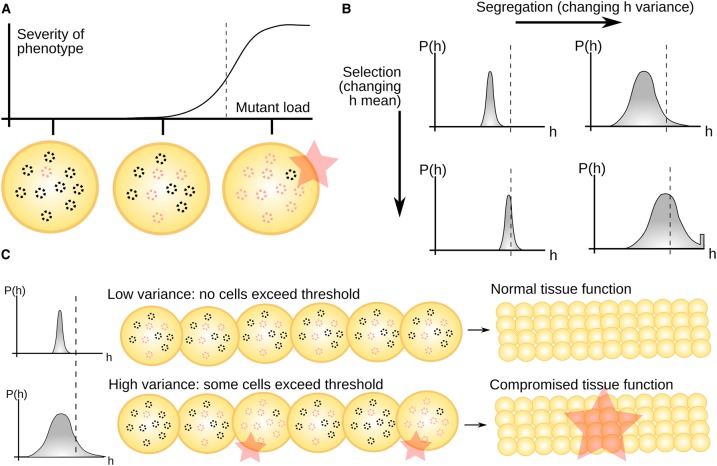
Thresholds in mtDNA mutant load. (**A**) The severity of symptoms associated with a pathogenic mtDNA type (red) is low or negligible until a mutant load threshold value (dashed line) is crossed, whereupon the disease severity increases dramatically [73]. (**B**) Influence of cellular processes on cell-to-cell mutant load distributions (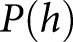 is the probability of a cell having a given mutant load 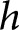). Segregation widens mutant load distributions; selection shifts their mean. Wider distributions have more probability of crossing mutant load thresholds (dashed lines). (**C**) Even a small number of high-mutant-load, dysfunctional cells can compromise organ-wide functionality [74,75]. If the cell-to-cell variance of heteroplasmy is low, few cells will cross the threshold and the organ can function as normal. If the cell-to-cell variance is high, even with a low mean mutant load, more cells will cross the mutant load threshold and disease will be manifest. We therefore need to understand (at least) both the mean and the cell-to-cell variance of heteroplasmic cells.

Some sequence variants may compromise bioenergetic functionality. However, because of the many copies of mtDNA in each cell and some redundancy, these variants typically need to be present above a certain mutant load in order to have a detrimental effect (Figure 2A) [71,72]. This is the so-called threshold effect in mitochondrial disease [73].

The threshold effect means that cell-to-cell differences in mtDNA mutant load are important. Imagine that an organism carries a mutant at an average 50% mutant load. If all cells are identical, none will pass a 60% threshold for the disease. However, if substantial cell-to-cell variability exists, some cells may exceed the disease threshold (Figure 2B). This potential for threshold crossing is important because of another nonlinearity. Some tissues require the concerted functionality of many cells working together. Just a small number of compromised cells can then lead to a pathology (Figure 2C). The presence of mitochondrially compromised cells has been shown to cause pathologies including arrhythmias in the heart [74] and damage in muscle fibres [75].

As this mini review will argue, these nonlinearities mean that it is important to study at least both the mean and the cell-to-cell variance of mutant statistics in mtDNA populations (and ideally the full distributions [58,76,77]). Changes to either can lead to pathological situations (Figure 2B) and both have consequences in the fundamental biology of inheritance and evolution [27,35,60]. In this mini review, we will highlight some of the several classes of biological process that alter these statistics of cellular mtDNA populations over time. We will focus on selection and segregation, respectively, changing the mean and variance of mutant mtDNA statistics. Due to length constraints, we can only briefly mention recombination, *de novo* mutations, and intercellular mitochondrial transfer, other processes which impact mtDNA populations in cells.

Within this scope, the big questions are: under what circumstances (organism-tissue-mtDNA sequence-time) does mtDNA selection pressure act? And how is cell-to-cell variability generated in mtDNA populations?

## Changing mean heteroplasmy

Perhaps the most dramatic process influencing cellular mtDNA populations in many sexually reproducing organisms is the clearing of mtDNA from one parent (usually paternal). This clearance strongly diminishes or removes mtDNA content from one parent around fertilisation, avoiding admixtures of maternal and paternal mtDNA. Postulated reasons for this clearance include the general exclusion of any foreign DNA from the fertilised oocyte, the avoidance of nuclear-mtDNA or mtDNA-mtDNA incompatibilities, and the mitigation of selfish mtDNA behaviour (reviewed in [59,78]). However, the search for a universal explanation is complicated by the diverse modes of mtDNA inheritance across life [27,61,79–82]. While maternal mtDNA inheritance is common, some organisms display parental or doubly unipaternal inheritance (DUI), and rare mtDNA ‘leakage’ (for example, rare retention of limited paternal mtDNA) can retain some heteroplasmy.

Animal mtDNA inheritance is usually maternal [27,61], with some exceptions including bivalves adopting DUI [83,84], and paternal leakage sometimes reported (and highly debated) in humans [85,86]. Plants usually inherit mitochondria maternally, with exceptions including paternal leakage [87], inheritance, or DUI in some species [88–91]. Fungal mtDNA inheritance is more complex and different species may undergo uniparental inheritance and/or DUI [26,27] and can also involve the inheritance of mitochondrial ‘plasmids’ [92].

If heteroplasmy exists after fertilisation (for example, due to leakage or mutation), cellular processes may change heteroplasmy statistics over time (Figure 1). In developing animals, heteroplasmy changes in a tissue-, sequence-, and time-dependent way [93]. Animal models have allowed increasingly detailed insight into these dynamics. Typically, a model is constructed or acquired harbouring an admixture of two mtDNA types, and techniques including pyrosequencing, qPCR, and dPCR are used to compare heteroplasmy in aged organisms against some reference. Mouse models have been particularly well explored here, including a widely used pairing of C57BL/6 or BALB and NZB [94–96], other pairings [97,98], and the more recent construction of admixtures with a range of genetic distances between the interrogated haplotypes [99,100]. Fly [101,102] and livestock [103,104] models have also been investigated. Recent advances in these model systems have included minipigs [105], and an elegant system in *Drosophila* allowing different modes of mtDNA selection to be characterised [106].

The ongoing development of diverse models has underlined that selection, leading to systematic changes in mean heteroplasmy, is common among mtDNA pairings. Within a pairing, one type may experience an advantage in some tissues and a disadvantage in others. Selective differences are often particularly pronounced in liver, spleen, kidney, and blood (observed in most references above), but are manifest in many tissues, including post-mitotic tissues including brain, heart, and muscle [99,105]. For some pairings, we have found that selective differences depend on time and developmental stage [99]. The expansion of mouse models has suggested that the magnitude of these selective differences may be related to the genetic diversity of the mtDNA pairing, with more diverse pairs showing stronger differences [99] and similar pairs showing little difference [100]. However, *in vitro* results from human oocytes and oocyte-derived material have challenged this picture, showing little relationship between heteroplasmy shift and genetic diversity [107,108] (see corrected data [109] for Ref. [108]). The mapping between these *in vitro* results, with associated passage protocols, culture conditions, etc., and natural development is not yet completely straightforward. However, a potential reconciliation of all approaches involves viewing selective differences as resulting from a combination of genetic features; more diverse molecules have a higher probability, but not a necessity, of differing at these features. The substantial mtDNA diversity present in human populations suggests that selective differences may be common in pairings arising from gene therapies [110].

Individual-level mtDNA selection is observed in disease-causing human mutations. MtDNA carrying the 3243A
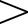
G mutation, for example, is depleted over time in leucocytes [111]. Notably, the presence of the 3243A
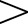
G mutation affects overall cellular mtDNA copy number, perhaps via a compensatory mechanism aiming to maintain a given wild-type content [112,113].

Selection in the germline has proved more controversial, due in part to the lower magnitudes of selective difference observed. Studies on mammalian germline development have shown that the development of oocytes and development post-fertilisation can show different patterns of selection. Several studies in mice [114] and human [115,116] found random drift to explain heteroplasmy distribution in oocytes. However, selection has been observed to act on these random oocyte distributions before, or during, their development to offspring [117–119]. Selection acting on deleterious human mutations, for example, the 3243A
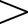
G mutation above, has been suggested in germline development [120,121], and a recent large-scale study has found evidence for germline selection, under nuclear control, at different mtDNA loci [122]. To dissect the dynamics of germline mtDNA selection, we recently described mtDNA dynamics during development and between generations in two mouse models with different mtDNA pairings [123]. One showed selection for mtDNA content in oocytes that was subsequently reversed in transmission to pups; the other contrasting case showed no selection apparent in oocytes, but a clear selective difference was found in pups. This work both revealed mammalian germline mtDNA selection and identified haplotype-specific timing differences in its manifestation [123].

In plants, the diversity of naturally occurring mtDNA forms supports a wider range of dynamic behaviour. Heteroplasmy in mtDNA structure, as well as sequence exists, perhaps reflecting a functional difference between large and small/absent molecules and their corresponding organelles [7,39–42,124–126]. Some plants seem to maintain a relatively simple tripartite system of one large and two smaller mtDNA molecules [125,127]. Others partition their genome into dozens of different ‘chromosomes’ [22,128]. Some structural variants are present at very low copy numbers, 10–1000 times lower than the dominant genomes [52], so that sometimes only a small fraction of plant cells contains these so-called ‘sublimons’ [129]. Of particular note is substoichiometric shifting, where sublimon mtDNA types at initially low copy number are rapidly elevated to dominate mtDNA populations [130,131]. These fast heteroplasmy shifts often have dramatic phenotypic consequences including cytoplasmic male sterility (CMS) [55,132–134], where the ability to produce functional pollen, anthers, or male gametes is compromised. CMS has been observed naturally in over 150 species [135]. This is detrimental for the plant but of profound use in crop breeding, allowing the easy construction of productive hybrids [134,136], increasing crop production in an increasingly challenged world [137].

In fungi, a history of literature has considered competition between ‘petite’ mutants where mtDNA suffers a deletion (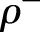) or is absent (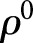) and wildtype (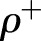) in single yeast cells [138,139]. Selfish replication is often observed, where small mtDNAs with relatively many origins of replication outcompete longer mtDNAs [140]. The magnitude of this advantage can be changed by modulating the functional challenges that the cell’s mitochondria face [141].

These species-, sequence-, tissue-, and time-dependent observations mean that the circumstances under which selection acts on mtDNA populations (i.e. inducing a systematic, reproducible change in mean heteroplasmy) remain unresolved. How are these different dynamics manifest at the molecular level? Several possible mechanisms for selective differences likely compete [142]. In several systems, ‘selfish’ behaviour of molecules with an intrinsic replicative advantage has been found [78,143,144]. These include deletion mutants in nematodes [145], short molecules with high replication origin density in yeast [140] and plants [146], and possibly particular D-loop variants in humans [108,147,148].

Features beyond replication rate may also influence mtDNA selection. Some nuclear-encoded factors influencing segregation have been identified [149]. Mitochondrial quality control [56] acts to remove poorly performing organelles, which may have a selective effect if different mtDNA types vary in metabolic or bioenergetic function. Differences in oxidative phosphorylation exist between human haplogroups [150] and in reactive oxygen species production in mouse strains [151]. Some evidence exists for the magnitudes of selective differences being linked to the turnover rate of mtDNA in cells (or cells themselves) [99]. Environmental pressures may provide further selective pressures. Although association studies with mtDNA are challenging [152,153], evidence in fish suggests that mtDNA variants have been shaped by local climate [154], and environmental effects on human mtDNA have been reported [155] including a role for altitude [156] and temperature [157].

## Changing heteroplasmy variance

In parallel with changing mean heteroplasmy, the cell-to-cell variance in heteroplasmy is also changed by several biological processes. Typically, changes in variance are harder to detect than changes in mean, and the large uncertainties involved are often ignored [158]. This is because limited sampling challenges estimates of variance, measurement noise can confound observations of variance, and averaging across cells (as in, for example, amalgamated tissue samples) loses information on cell-to-cell variance.

In animals, a developmental ‘genetic bottleneck’^1^ increases cell-to-cell heteroplasmy variance from the fertilised oocyte (which, as a single cell, has zero variance) [159,160]. One purpose of this process appears to be to generate heteroplasmy variance between oocytes in the next generation. Cells carrying low levels of pathogenic mutations can then be fertilised and those carrying high levels can be discarded, overcoming Muller’s ratchet [161] via cell-level selection.

The genetic bottleneck was originally found in cattle [162,163] and has since been demonstrated in animals from mice [160,164–166] and salmon [167] to humans [168–172]. The mechanism of the genetic bottleneck remains debated [160,164–166]. A physical bottleneck, involving a reduction in cellular mtDNA copy number during germline development, occurs in several animals [164,166,170,173,174]. This physical bottleneck likely plays a role both through the amplification of genetic drift and variability induced from mtDNA population processes (Figure 1) but is not equivalent to the genetic bottleneck [160,175]. Other processes generating mtDNA variability — that may be amplified by the physical bottleneck — include random turnover due to stochastic mtDNA replication and degradation [45,47,164], (related) participation of a random subset of mtDNA molecules in replication [166], and random partitioning of individual mtDNAs [164], or clusters of mtDNA molecules [165] at cell divisions. Using all available experimental data from mice, and new experiments, we used an unbiased approach to compare these mechanisms and found that random turnover and binomial partitioning (BDP or birth-death-partitioning) was the most supported mechanism [160].

In plants, germline development is complex and debated [176,177]. Different modes of inheritance are observed (paternal, maternal, biparental) for mitochondria (and plastids) in different species [87,88,91]. MtDNA variance certainly exists, with a suggestion of a ‘bottleneck’ in plants made after observing mean and variance changes after two generations of sexual reproduction [68]. Tissue variability in subgenomic mtDNA molecules has been reported [178] and is predicted to arise from random mtDNA dynamics [146,179].

In fungi, segregation of mtDNA at cell divisions was reported in the 1970s [180], and the interplay of segregation, recombination, and uniparental inheritance in increasing or stabilising mtDNA variance has been explored since [181,182]. In yeast, tighter control (i.e. closer to perfect halving than random binomial sampling) of mtDNA partitioning has been demonstrated [183], limiting but not removing mtDNA variability.

**Figure 3.**
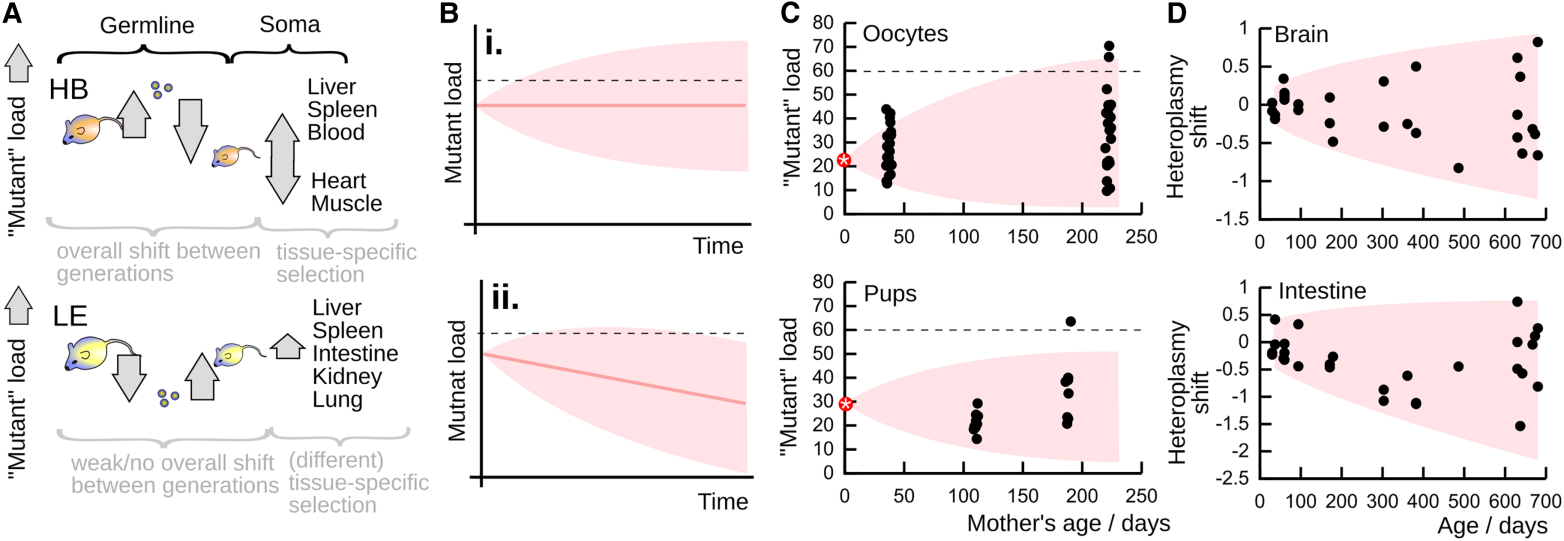
Changes in heteroplasmy mean and cell-to-cell variance in germline and somatic tissues. Mouse models consisting of wild-derived mtDNA haplotypes in cellular admixture with the C57Bl/6N haplotype have recently demonstrated diverse mtDNA behaviour [93,123]. Here, HB and LE are two different mouse models, consisting of an admixture of wild-derived mtDNA (either haplotype HB or LE, referring, respectively, to Hohenberg and Lehsten, the localities where the original wild mice were captured) with C57BL/6N mtDNA. (**A**) The HB model shows an overall germline mean shift manifest by an increase in oocyte mutant load and an overcompensatory decrease in pup mutant load, followed by the somatic selection that shows tissue-specific variation in direction. The LE model, like other mouse models, shows little overall germline shift but does show an inverted decrease in oocyte mutant load matched by a compensatory increase in pup mutant load. LE also shows tissue-specific selection during development, in different patterns to HB. (**B**) We generally find an increase in cell-to-cell mutant load variance over time (i). Even when coupled with a mean decrease in mutant load (ii), an increase in variance can still lead to threshold crossing. (**C**) Increasing variance in HB oocytes and pups, from Ref. [123]. The increased spread of mutant load values over time (sketched in shaded regions) leads to oocytes and pups from older mothers (with comparable initial heteroplasmies, red stars) crossing thresholds. (**D**) Increasing mtDNA heteroplasmy variance in many-cell samples from the brain (low cell turnover) and intestine (high cell turnover) from Ref. [93].

During organismal ageing, and in somatic tissues, the variance of mtDNA populations also increases over time.^2^ Re-analysis of data from Ref. [93] shows increasing variance even in tissue-averaged samples in mouse brain (slow cell turnover) and intestine (fast cell turnover) (Figure 3). Somatic, tissue-specific increases in heteroplasmy variance have been inferred during human embryogenesis using a powerful population phylogenetic approach [172]. In the mouse germline, we recently showed that cell-to-cell variance continues to increase as mothers age [123]. This observation supports theoretical modelling [48] and re-analysis of earlier results from fly [102] and mouse [166] (in Ref. [48]).

In humans, increasing heteroplasmy variance — via the mtDNA bottleneck and other processes — has the effect of complicating clinical planning for inherited diseases, because the mutant load inherited by a given child is a random variable. The increase in heteroplasmy variance between generations is different for different mtDNA mutations [184]. A striking example is the fast shifts towards homoplasmy for 8993T
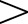
C/G mutations compared with the slower increase in variance associated with 3243A
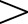
G [120,184]. In human cell lines harbouring the 3243A
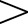
G mutation, a variety of outcomes exists, reflecting either direction of drift or comparative stability, perhaps modulated by nuclear genes [185,186].

## Mutations and intercellular transfer

While not a focus of this mini review, we briefly note that the appearance and physiological influence of *de novo* mutations in evolving mtDNA populations has been a matter of some debate. Redox imbalance is hypothesised to be an important source of DNA damage [187,188]. However, the link between mitochondrial redox activity and mutation is not uncontroversial. In some experiments, more severe oxidative damage did not dramatically influence mtDNA mutation rates [189], and mtDNA mutational profiles suggest that other sources like replication errors may be more important [190,191]. Regardless of generative mechanism, in humans, *de novo* point mutations are a common cause of mtDNA disease [192].

To dissect the role of mtDNA mutations in physiology, the elegant ‘mutator mouse’ system has been developed, where defective mtDNA polymerase leads to the accumulation of mtDNA mutations over time [193]. These mice show severe phenotypes reminiscent of ageing [193], though the inference that these phenotypes provide a causative link between mtDNA mutations and ageing has been debated [194].

Another way in which cellular mtDNA populations can change is through the ‘immigration’ of mitochondria from external sources. Horizontal transfer of mitochondria between cells has been reported in a variety of (often pathological) circumstances, and through a range of mechanisms including tunnelling nanotubes, extracellular vesicles, gap junctions, and cell fusion (reviewed in [195–197]). Several studies have shown that, in cells lacking mtDNA, external acquisition of mtDNA rescues depleted respiratory function and tumorigenic potential [198,199]. In accordance with stochastic theory [57,58], this external ‘immigration’ of mtDNA can stabilise heteroplasmy distributions that may otherwise be unstable [200].

## Theory

The analogy of organisms in an ecosystem translates through to several ideas from population genetics that have been used to describe the dynamics of mtDNA populations. Approaches from statistical genetics (i.e. focussing on summary statistics of populations) [76,201,202], stochastic modelling (i.e. considering the influence of random processes on populations of molecules) [45,47,48,58,146,160,179], and simulation (i.e. computational representation of mixed or spatially distributed molecules) [203–206] have been proposed and recently reviewed in ref. [207]. A theory has been proposed describing the stochastic behaviour of general physical organelles [208] and associated steady-state [77] and time-dependent [58] distributions have been calculated. Stochastic approaches specific to mtDNA have characterised changes in heteroplasmy mean [99] and variance [123,160], identified the general prediction that heteroplasmy variance increases linearly with time and mtDNA turnover [48], described the capacity for cellular control on mtDNA [48,209], elucidated recombination dynamics in plants [146,179], revealed links between physical and genetic mitochondrial dynamics [203–205,207], and dissected variability arising from natural and experimental sources [158,206].

Some straightforward insights from this body of theory can help increase the power and reliability of studies on heteroplasmy. First, it must be remembered that the analysis of percentage point differences in mutant load (e.g. labelling a change of 50% to 60% as 10 percentage points) has several limitations when analysing mtDNA data. Under the same selective pressure, the mutant load will change by different amounts depending on its initial value (for example, a change of 10 percentage points from 50 to 60% is very possible, but a change from 95% to 105% is not). Heteroplasmy changes across samples with different starting values are therefore not immediately comparable. Mathematical theory motivates a simple transformation [48,99], reflecting the difference in fitness between two mtDNA types [94,101], that accounts for this and allows heteroplasmy readings at different levels to be compared:(1)
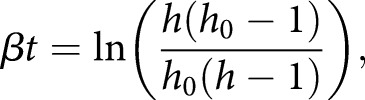
where 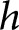 is an observed ‘final’ mutant load, 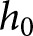 is a reference ‘initial’ mutant load, and 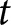 (if known) is the time between these measurements. 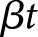 reflects a selective difference 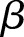 acting over a time 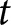, arising from the mathematical prediction that mean mutant load will evolve through sigmoidal dynamics according to:(2)
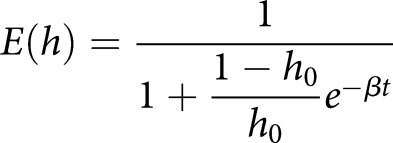
This representation fails in homoplasmic situations (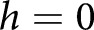 or 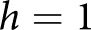); including homoplasmy requires a more detailed distributional picture (see below).

Predictions of heteroplasmy variance are challenging in the face of selection. For neutral mtDNA evolution and no cell divisions, a detailed, stochastic, microscopic model of mtDNA dynamics predicts that cell-to-cell mutant load variance 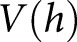 (linked to the widths of the distributions in Fig. 2) increases linearly with time 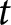 [48]:(3)
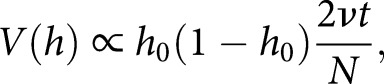
where 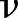 is the rate of mtDNA turnover and 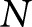 the size of the cellular mtDNA population. The constant of proportionality is predicted in recent work to be 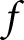, the fraction of fragmented mitochondria (i.e. those subject to degradation) [50]. Variance increase due to cell divisions [210] can also readily be included via an additional term in equation (3) [48]. This linear increase in 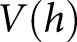 is compatible with our recent experimental observations above [123,160]. Previous work often uses expressions including 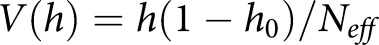 or 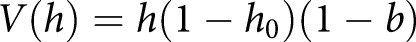 to define an effective ‘bottleneck parameter’ 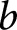 [76] or ‘bottleneck size’ 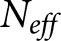 (found in several studies based on a binomial sampling model of the bottleneck). Equation 3 allows us to start linking these effective quantities (which, as above, do not directly correspond to observable numbers of molecules) to real biological measurements 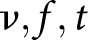, and 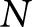.

Under neutral conditions (no systematic selection), the Kimura distribution has been proposed as a model for cell-to-cell distributions of mutant load [76]. This has advantages over normal and binomial alternatives, although it must be remembered that a fit to a Kimura distribution does not necessarily provide evidence against selection: an mtDNA population where the mean heteroplasmy has changed over time may still conform to a Kimura distribution. A truncated Kimura distribution has been proposed to include one mode of selection in distributional calculations, by disallowing mutant load values above a given cutoff [121]. The full distributional solutions for mtDNA populations that may be under selection, undergo cell divisions, and systematically change population size through a physical bottleneck have been derived [160], though as these are complex, a more heuristic combination of a truncated normal distribution accounting for homoplasmy has been heuristically used [160].

Another branch of mtDNA modelling has addressed evolutionary questions, including the interplay between mtDNA and the evolution of sex [211] and uniparental inheritance [212], recombination strategies [146,179,213], and the emergence of a distinct germline [214]. We recently used a modelling approach to reveal the features governing mtDNA gene loss across life [10] and to propose a hypothesis for the differences between plant and animal mtDNA structure and dynamics based on the immobility of plants [7].

## Conclusions

This mini review has argued that both mean and cell-to-cell variance, and ideally full distributions, of mtDNA mutant load are important to understand both for basic science and clinical planning. Heteroplasmy variance can lead to pathological thresholds being exceeded even for populations with lower mean mutant load and provides an important source of cell heterogeneity both within and between generations. Ongoing progress in characterising the processes that affect the cell-to-cell variance of mtDNA populations is highly desirable.

The expansion of available animal models, in conjunction with developing theory, is increasing our knowledge of the diverse ways that mtDNA populations change over time. One recent example is coupled experimental evidence [123] and theoretical support [48,50] for a linear increase in heteroplasmy variance over time during ageing.

An expansion of theory that is able to describe the mean and variance (and distributional details) of mtDNA populations under selection will improve our ability to characterise mtDNA populations. Currently, several common analytical approaches are not robust to even small selective differences. The field will in future benefit from an expansion of the available mtDNA pairings that can be considered in biological models, which will increase our ability to identify and verify the genetic features governing these biologically and medically important shifts in mtDNA population structure.

**Perspectives**

- Evolving mtDNA populations within cells are vital across eukaryotic life, from plants and fungi to humans. How they change with time underlies fundamental biology and translational bioenergetics, from inherited diseases to crop sterility. Nonlinear links between mutant load and cellular phenotype mean that it is important to understand both the cell-to-cell mean and variance (and ideally the full distributions) of mtDNA populations.
- Model organisms and increasingly high-resolution technology provide valuable insight into the dynamics of mtDNA populations, but many mechanistic questions remain. This is particularly true in non-mammalian organisms, where mtDNA dynamics can be much more complex (for example, mtDNA recombination in plants). The organism-, tissue-, sequence-, and time-dependent features that cause changes in mtDNA population structure remain poorly understood.
- More diverse biological models, in tandem with more developed quantitative theory, will in future help to reveal the mechanisms shaping these essential populations.

## Footnotes

^1^When discussing ‘the bottleneck’ it is important to be clear about which process is being referred to. The ‘genetic bottleneck’ is a model describing how much heteroplasmy variance is generated over a given period, but it corresponds to an ‘effective’ quantity that does not reflect a given observable number of mtDNA molecules [48,79]. There is also a ‘physical bottleneck’, a specific observable depletion of mtDNA copy number, that contributes to, but is not identical to, the genetic bottleneck.

^2^Here, we avoid the term ‘genetic bottleneck’ because of its aforementioned potential confusion with mtDNA population size reduction.

## Abbreviations

CMS: cytoplasmic male sterility
DUI: doubly-unipaternal inheritance
mtDNA: mitochondrial DNA
